# Population pharmacokinetics of TLD-1, a novel liposomal doxorubicin, in a phase I trial

**DOI:** 10.1007/s00280-024-04679-z

**Published:** 2024-06-15

**Authors:** Anna M. Mc Laughlin, Dagmar Hess, Robin Michelet, Ilaria Colombo, Simon Haefliger, Sara Bastian, Manuela Rabaglio, Michael Schwitter, Stefanie Fischer, Katrin Eckhardt, Stefanie Hayoz, Christoph Kopp, Marian Klose, Cristiana Sessa, Anastasios Stathis, Stefan Halbherr, Wilhelm Huisinga, Markus Joerger, Charlotte Kloft

**Affiliations:** 1 Department of Clinical Pharmacy and Biochemistry, Institute of Pharmacy, Freie Universitaet Berlin, Kelchstr. 31, 12169 Berlin, Germany; 2 Graduate Research Training Program PharMetrX, Freie Universitaet Berlin/University of Potsdam, Berlin/Potsdam, Germany; 3 Department of Medical Oncology and Haematology, Cantonal Hospital St. Gallen, Rorschacher Strasse 95, 9007 St. Gallen, Switzerland; 4 Department of Medical Oncology, Oncology Institute of Southern Switzerland, EOC, Bellinzona, Switzerland; 5 Department of Medical Oncology, Inselspital Bern University Hospital, University of Bern, Bern, Switzerland; 6 Department of Medical Oncology, Kantonsspital Graubünden, Chur, Switzerland; 7 Oncology/Hematology, Kantonsspital Graubünden, Chur, Switzerland; 8 Coordinating Center, Swiss Group for Clinical Cancer Research, Bern, Switzerland; 9 Oncology Institute of Southern Switzerland, EOC, Bellinzona, Switzerland; 10 Faculty of Biomedical Sciences, Universita della Svizzera Italiana, Lugano, Switzerland; 11 Innomedica Switzerland AG, Bern, Switzerland; 12 Institute of Mathematics, University of Potsdam, Potsdam, Germany

**Keywords:** Nanoparticles, Liposomes, Doxorubicin, Pharmacokinetics, Pharmacometrics, Nonlinear mixed-effects model

## Abstract

**Study objectives:**

TLD-1 is a novel pegylated liposomal doxorubicin (PLD) formulation aiming to optimise the PLD efficacy-toxicity ratio. We aimed to characterise TLD-1’s population pharmacokinetics using non-compartmental analysis and nonlinear mixed-effects modelling.

**Methods:**

The PK of TLD-1 was analysed by performing a non-compartmental analysis of longitudinal doxorubicin plasma concentration measurements obtained from a clinical trial in 30 patients with advanced solid tumours across a 4.5-fold dose range. Furthermore, a joint parent-metabolite PK model of doxorubicin_entrapped_, doxorubicin_free_, and metabolite doxorubicinol was developed. Interindividual and interoccasion variability around the typical PK parameters and potential covariates to explain parts of this variability were explored.

**Results:**

Medians $\pm$ standard deviations of dose-normalised doxorubicin_entrapped+free_ C_max_ and AUC_0−∞_ were 0.342 $\pm$ 0.134 mg/L and 40.1 $\pm$ 18.9 mg·h/L, respectively. The median half-life (95 h) was 23.5 h longer than the half-life of currently marketed PLD. The novel joint parent-metabolite model comprised a one-compartment model with linear release (doxorubicin_entrapped_), a two-compartment model with linear elimination (doxorubicin_free_), and a one-compartment model with linear elimination for doxorubicinol. Body surface area on the volumes of distribution for free doxorubicin was the only significant covariate.

**Conclusion:**

The population PK of TLD-1, including its release and main metabolite, were successfully characterised using non-compartmental and compartmental analyses. Based on its long half-life, TLD-1 presents a promising candidate for further clinical development. The PK characteristics form the basis to investigate TLD-1 exposure-response (i.e., clinical efficacy) and exposure-toxicity relationships in the future. Once such relationships have been established, the developed population PK model can be further used in model-informed precision dosing strategies.

**Clinical trial registration:**

ClinicalTrials.gov–NCT03387917–January 2, 2018

**Supplementary Information:**

The online version contains supplementary material available at 10.1007/s00280-024-04679-z.

## Introduction

### Efficacy and safety of doxorubicin

Doxorubicin is a well-established and highly efficacious drug used for the treatment of several tumour entities such as breast cancer, ovarian cancer, Kaposi’s sarcoma, lymphoma, and multiple myeloma [1–3]. The mechanism of action of this anthracycline is a combination of free radical formation, cellular membrane interaction, topoisomerase II inhibition, and DNA intercalation, all ultimately leading to apoptosis [1, 3, 4]. Unfortunately, only a small fraction of doxorubicin accumulates in the tumour, resulting in high drug exposure in healthy tissue and associated toxicities [5]. Degradation of doxorubicin in the blood stream and absorption and accumulation of the formed reactive oxygen species into the heart tissue leads to cardiomyocyte apoptosis and irreversible cardiac damage [2]. This cardiotoxicity of doxorubicin is further amplified by the cardiotoxic effect of doxorubicinol, which is the primary circulating metabolite of doxorubicin [2, 6, 7]. Due to its irreversible cardiac damage, doxorubicin is only given until a pre-determined cumulative lifetime dose is reached, often resulting in early termination of the otherwise efficacious treatment [2, 5].

### Benefits, challenges, and pharmacokinetics of pegylated liposomal doxorubicin

Entrapping doxorubicin into polyethylene glycol modified (PEGylated) liposomes (such as in Caelyx^®^ (Europe) /Doxil^®^ (US), hereinafter referred to as Caelyx^®^) [8] largely reduces the observed cardiotoxicity [5] by increasing drug accumulation and release at the tumour site by approximately 10-fold [9]. Due to their large molecular size, the liposomes exploit the enhanced permeability and retention effect (EPR) [10, 11] by only extravasating into tissue with increased vascular permeability, such as tumours [12]. Thus, this targeted drug delivery strategy strongly increases the antitumour effect while simultaneously reducing the adverse effects in healthy tissue. Caelyx^®^ is approved for the treatment of several tumour entities, such as ovarian cancer, breast cancer, myeloma, and Kaposi-Sarcoma [8]. However, approximately 50% of patients treated with Caelyx^®^ suffer from palmar-plantar erythrodysaesthesia (PPE) [5, 13], a painful inflammation of the palms of the hands and soles of the feet. Furthermore, approximately one in four patients experiences mucositis [5].

The clinical pharmacokinetics (PK) of PEGylated doxorubicin are significantly different from the PK of free doxorubicin [3, 4, 10, 14] and characterised by a small volume of distribution [8, 15], a low clearance, a long half-life of 50–80 h, and an approximately 300-fold higher area under the concentration-time curve (AUC) [10]. The drug effect of PEGylated liposomal doxorubicin is associated with the liposome-released unbound (free) concentration of the parent drug doxorubicin and to a lesser extent with its main metabolite doxorubicinol in the tumour cell; however, the PK of liposome-entrapped doxorubicin and its interplay with the two free species has not been well studied [10, 20]. Due to their long circulation time, it is hypothesised that the doxorubicin-containing liposomes extravasate into and accumulate in the skin at the pressure points of the hands and the feet, and that this accumulation is a key factor in the development of PPE [10, 16, 17].

### TLD-1, a novel pegylated liposomal doxorubicin

To maximise the efficacy and minimise the toxicity of PEGylated liposomal doxorubicin, TLD-1, a novel PEGylated liposomal doxorubicin formulation [18], is currently under investigation. Compared to Caelyx^®^, TLD-1 consists of smaller, uniform, and more stable liposomes with an average diameter of 36 nm (average diameter of Caelyx^®^: 70 nm) [18]. TLD-1 is being developed to optimise the antitumour activity-toxicity ratio of PEGylated liposomal doxorubicin, and preclinical studies suggest a potential for an improved safety profile, including the lack of PPE in animal models. In the dose escalation part of the first-in-human phase I clinical trial including 12 patients (NCT03387917), grade 2/3 cumulative PPE was observed in four patients [19]. Moreover, two patients experienced grade 2 mucositis and further two patients experienced grade 2 rash [19].

### Objectives

In this work, we aimed to characterise the PK of TLD-1 using non-compartmental analysis and nonlinear mixed-effects modeling to jointly evaluate the pharmacokinetics of entrapped doxorubicin, free doxorubicin, and the main metabolite doxorubicinol in patients with advanced solid tumours. Typical PK values along with interindividual and interoccasion variability (i.e., between-patient and between-cycle variability) for the key kinetic processes, such as liposomal release and doxorubicin elimination, were estimated. Additionally, patient characteristics (covariates) influencing pharmacokinetic parameters were explored.

## Materials and methods

### Clinical study

The open-label, single-arm, multicentre, first-in-human phase I TLD-1 dose-escalation trial SAKK 65/16 (NCT03387917) in patients with advanced solid tumours was conducted at four phase I centres in Switzerland. The compound was administered via intravenous infusion in 21 days cycles for a maximum of 6 cycles for patients previously treated with anthracyclines or 9 cycles for patients previously not treated with anthracycline until disease progression, unacceptable toxicity, or withdrawal of consent. The infusion durations were 60 min for dose levels 1–6 (10–40 mg/m^2^) and 90 min for dose level 7 (45 mg/m^2^). Dose escalation followed an accelerated titration design until first occurrence of a dose-limiting toxicity. Afterwards, a continual reassessment method using cohorts of three was applied [21, 22]. Individual doses were based on one of seven dose levels and the individual body surface area (BSA) (Table 1). Upon treatment of 12 patients, dose level 7 was identified as the tentative maximum tolerated dose and nine additional patients were treated at this dose level. Due to several late appearing cumulative toxicities in this expansion cohort, nine additional patients were treated at dose level 6 (40 mg/m^2^).

**Table 1:** Dose levels 1–7 of TLD-1 used in the first-in-human phase I clinical trial SAKK 65/16

Dose level	Dose [mg] per m^2^ body surface area
1	10
2	16
3	23
4	30
5	35
6	40
7	45

Concentrations of total doxorubicin (doxorubicin_entrapped+free_), unencapsulated doxorubicin (doxorubicin_free_), and the main metabolite doxorubicinol were measured by Swiss BioQuant AG (Reinach, Switzerland) using a validated liquid chromatography coupled to tandem mass spectrometry (LC-MS/MS) method as described below. Samples were collected in the first two cycles at the following pre-defined time points: prior to infusion (t = 0), mid-infusion (0.5 h for dose level 1–6 and 0.75 h for dose level 7), end of infusion (1 h for dose levels 1–6 and 1.5 h for dose level 7), as well as, 0.5 h, 1 h, 3 h, 5 h, and 7 h after end of infusion, at 24 h, 48 h (only in cycle 1), 168 h (day 8), and 336 h (day 15).

### Bioanalytical method and assay performance

For the analysis of doxorubicin_free,_ doxorubicinol, and doxorubicin_entrapped+free,_ two validated bioanalytical assays were used.

Sample preparation for the quantification of doxorubicin_free_ and doxorubicinol was performed in an ice-bath and under light protected conditions. To an aliquot of 50 µL matrix, 100 µL of PBS buffer with 1.0% BSA containing the internal standard was added. After gentle shaking and storage for 5 min, the samples were transferred to an ultrafiltration tube and filtrated for 15 min at approximately 10,000*g*. The temperature of the centrifuge was set to 8 ℉C. An aliquot of 50 µL of the filtrate was transferred to an Eppendorf tube and precipitated with 100 µL of acetonitrile. After vortex mixing, the samples were centrifuged for 5 min at approximately 50,000 *g*. The temperature of the centrifuge was set to 8 °C. An aliquot of the supernatant was transferred to an autosampler vial for subsequent HPLC-MS/MS analysis.

Sample preparation for the quantification of doxorubicin_entrapped+free_ was done in an ice-bath and under light protected conditions. To an aliquot of 50.0 µL human plasma, 200 µL of acetonitrile containing the internal standard were added. After mixing, the samples were centrifuged for 10 min at approximately 50,000 *g*. The temperature of the centrifuge was set to 8 °C. An aliquot of the supernatant was transferred to an autosampler tube for subsequent HPLC-MS/MS analysis.

For the quantification of doxorubicin_entrapped+free_, sample analysis was done by column separation using reversed-phase liquid chromatography followed by detection with triple-stage quadrupole MS/MS in the selected reaction monitoring mode. Chromatography was performed by gradient elution using acidified water and acetonitrile (ACN; 5–95%).

For the quantification of doxorubicin_free_ and doxorubicinol, sample analysis was done by column separation using reversed-phase liquid chromatography followed by detection with triple-stage quadrupole MS/MS in the selected reaction monitoring mode. Chromatography was performed by gradient elution using acidified water and acetonitrile (ACN; 5–95%). On-line solid phase extraction with a reversed-phase trapping column was used to further purify and concentrate the sample prior to MS/MS quantification.

For doxorubicin_free_, the descriptive statistics of the QC of the batch in the calibration range of 2.00–2000 ng/mL showed an inter-batch precision of 4.8–10.9%, whereas the inter-batch accuracy was in the range of 99.0–101.3% of the nominal concentration.

For doxorubicin_entrapped+free_, the descriptive statistics of the QC of the batch in the calibration range of 20.0–20,000 ng/mL showed an inter-batch precision of 3.2–6.3% whereas the inter-batch accuracy was in the range of 100.0–104.3% of the nominal concentration.

For doxorubicinol, the descriptive statistics of the QC of the batch in the calibration range of 0.500–500 ng/mL showed an inter-batch precision of 5.3–12.1%, whereas the inter-batch accuracy was in the range of 98.0–99.0% of the nominal concentration.

### Analysis dataset generation

For each PK sampling timepoint, the concentration of doxorubicin_entrapped_ was calculated by subtracting the measured concentration of doxorubicin_free_ from the concentration of doxorubicin_entrapped+free_. In total, 1870 concentration measurements were available (n = 624 for total doxorubicin, n = 623 for doxorubicin_free_, and n = 623 for metabolite doxorubicinol). For the one sample with missing doxorubicin_free_ and doxorubicinol concentrations, it was also not possible to derive the corresponding doxorubicin_entrapped_ concentration by subtracting the concentration of doxorubicin_free_ from the concentration of doxorubicin_entrapped+free_. Concentration measurements below the lower limit of quantification (BLOQ) (n = 314, all doxorubicinol) were removed from the analysis dataset. Based on in-house data showing that usually > 99% of doxorubicin was entrapped in liposomes in the final product, it was assumed that 100% of the doxorubicin was entrapped at time of infusion. Patient characteristics age, body weight, body height, and BSA, and clinical chemistry parameters serum creatinine, alanine aminotransferase, aspartate amino transferase, alkaline phosphatase, bilirubin, alkaline phosphatase, serum creatinine and the estimated glomerular filtration rate according to the CKD-EPI formula [23] were included in the dataset and available for testing as potential covariates during model development. Moreover, additional body size descriptors lean body weight [24] and body mass index (BMI) were calculated and available for testing as potential covariates.

### Non-compartmental analysis

Dose-normalised maximum concentration (C_max_), area under the concentration-time curve from t = 0 until infinity (AUC_0-∞_), and the terminal half-life of total doxorubicin (doxorubicin_entrapped+free_) were calculated for every patient and cycle using R Statistical Software [25] and the package *pkr*. Dose proportionality was assessed by inspecting dose-normalised C_max_ and AUC_0−∞_ vs. dose for trends. Due to the unbalanced number of patients in each dose level, no additional statistical tests were performed to assess dose proportionality [26], however, the possibility of a nonlinear clearance was further explored in the nonlinear mixed-effects analysis. A possible cycle-dependent clearance was investigated by inspecting the ratios of individual dose-normalised AUC_0−∞_ in cycles 1 and 2 for trends.

### Nonlinear mixed-effects pharmacokinetic model

A nonlinear mixed-effects modelling approach [27, 28] was chosen to develop a joint parent (entrapped-free)-metabolite model for TLD-1 consisting of three submodels: the structural submodel, characterising the typical concentration-time profile of doxorubicin_entrapped_, doxorubicin_free_, and metabolite doxorubicinol using ordinary differential equations, the stochastic submodel, characterising different levels of variability around PK parameters of the structural model and concentrations, and the covariate submodel, aiming to identify patient characteristics that explain parts of this variability.

Model development was performed using NONMEM^®^ Version 7.4 (Icon Development Solutions, Ellicott City, MD, USA). Model parameters were estimated using the First-Order Conditional Estimation with Interaction (FOCE+I) algorithm implemented in NONMEM. Relative standard errors (RSE) were obtained using the $COVARIANCE function. For the final model, sampling importance resampling (SIR) was additionally performed to assess parameter precision [29, 30]. SIR was chosen over the more common non-parametric bootstrap procedure since non-parametric bootstraps can result in incorrect confidence intervals when applied to small and heterogeneous datasets [31]. In contrast to the non-parametric bootstrap, SIR does not rely on resampling individuals in new datasets and is therefore more suitable for smaller datasets [31, 32].

#### Structural submodel

Based on the reported small volume of distribution of doxorubicin_entrapped_ approximating the plasma volume [8] and previous models for PEGylated liposomal doxorubicin [15, 33, 34], a one-compartment model was assumed for doxorubicin_entrapped_. Several elimination pathways, using (i) linear [15, 34], (ii) nonlinear [8], and (iii) parallel linear and nonlinear processes were investigated for doxorubicin_entrapped_ to characterise the release of doxorubicin from the liposomes and the degradation of the liposomes by the reticuloendothelial system (RES) [33, 34]. Based on previously published PK models for doxorubicin_free_, two- [4, 34] and three- [35–37] compartment models were investigated. Linear and nonlinear formation processes from doxorubicin_free_ to the metabolite doxorubicinol were investigated and, based on previous PK analyses, one- [4, 35, 36] and two- [37] compartment models were investigated for doxorubicinol. The respective best submodel was selected for each doxorubicin species based on PK parameter value plausibility, model fit, and parameter precision (RSE ≤ 30%).

#### Stochastic submodel

Interindividual variability (IIV) parameters were investigated on structural PK parameters using exponential functions and only retained if the inclusion improved model fit (as indicated by a decrease in the objective function value, an indicator of model fit, and in improved goodness-of-fit plots), parameter precision was adequate (RSE ≤ 50%), and inclusion did not lead to model overparameterisation as indicated by a condition number > 1000 [38]. Correlations between parameters were assessed and, if the correlation exceeded 0.8, a “shared-η approach” was used [28]. Interoccasion variability (IOV), for which the start of each cycle represented a new occasion, was next investigated for all parameters using exponential functions. Final IOV parameter selection followed the same criteria as the IIV parameter selection. To characterise the residual unexplained variability (RUV), additive, proportional, combined additive proportional, and log-transformed both sides approaches were investigated.

#### Covariate submodel

Potential patient characteristics to be implemented as covariates in the model were pre-selected based on plausibility, previous reports [36, 39], and availability in the dataset. Exploratory graphical analyses were used to assess potential trends between covariate values and PK parameter estimates. Continuous covariates (e.g., body surface area) were normalised to the respective median value of the study population and implemented using power relationships. Categorical covariates (e.g., sex) were implemented using fractional change models. Stepwise covariate modelling [40] using significance criteria of changes in the objective function value of 3.84 (α = 0.05, df = 1) for the forward inclusion and 7.88 (α = 0.005, df = 1) for the backward elimination were applied for final covariate selection. Furthermore, only precisely estimated (RSE: ≤ 30%) covariate effects were retained in the final model.

#### PK model evaluation

The final population PK model was evaluated using goodness-of-fit plots and plots showing observed concentrations overlaid with typical and individual model predictions. Systematic bias was assessed by plotting conditional weighted residuals (CWRES) vs. time and typical predictions. To assess predictive performance, a prediction-corrected visual predictive check (pcVPC, n = 2000 simulations) [41] was performed.

## Results

### Clinical data

Among the 30 patients included in the trial, the most frequent tumour types were breast cancer (43.3%), ovarian cancer (20.0%), and gastrointestinal cancer (3.3%). The remaining 33.3% of patients had other solid tumour types. In line with the high frequency of breast cancer and ovarian cancer, 80% of the patients were female. Median age and BSA were 67.5 years (range: 38–83 years) and 1.75 m^2^ (range: 1.44–2.44 m^2^), respectively. Median body mass index (BMI) and lean body weight were 24.7 kg/m^2^ (range: 16.5–42.2 kg/m^2^) and 48.5 kg (range: 38.7–77.6 kg), respectively. Clinical chemistry parameters were mostly within the normal range. The raw concentration-time data of all patients are shown in Supplementary Fig. 1.

### Non-compartmental analysis results

**Fig. 1:**
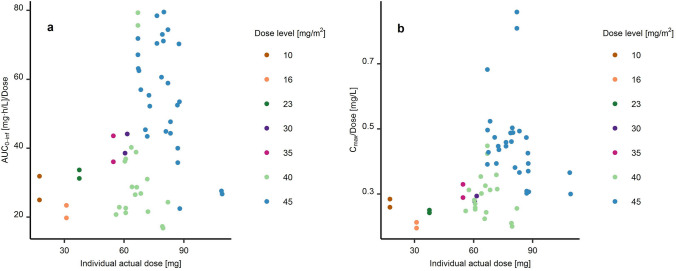
Dose-normalised AUC_0-inf_ (panal a, left) and dose-normalised C_max_ (panel b, right) vs. individual doses in the two first cycles of the 30 patients treated with TLD-1. *AUC*_*0-inf*_ AUC from t = 0 to infinity; *C*_*max*_ maximum concentration

For the non-compartmental analysis, two PK concentration-time profiles were not available, as no second cycle dose had been administered in two individuals (one in dose level 6 and one in dose level 7). Median dose-normalised AUC_0-∞_ and C_max_ of doxorubicin_entrapped+free_ were 40.1 h/L (range: 16.8–79.5 h/L) and 0.342 L^−1^ (range: 0.196– 0.859 L^−1^), respectively. The median half-life of doxorubicin_entrapped+free_ was 95 h with a large variability (range: 46–213 h). There was no clear relationship of increasing dose-normalised AUC_0−∞_ vs. dose and thus no clear sign of dose nonlinearity (Fig. 1).

However, due to the limited interpretability based on the unbalanced number of patients in each dose level, a potential nonlinear clearance was considered during the following compartmental model development. AUC_0-∞_ and half-life data for both cycles were available for 28 of the 30 patients. There was no trend for a cycle-dependent clearance, as indicated by the ratios of dose-normalised AUC_0−∞_ in cycle 2/cycle 1 being randomly scattered around 1 (Supplementary Fig. 2).

### Nonlinear mixed-effects pharmacokinetic model

#### Structural model

**Fig. 2:**
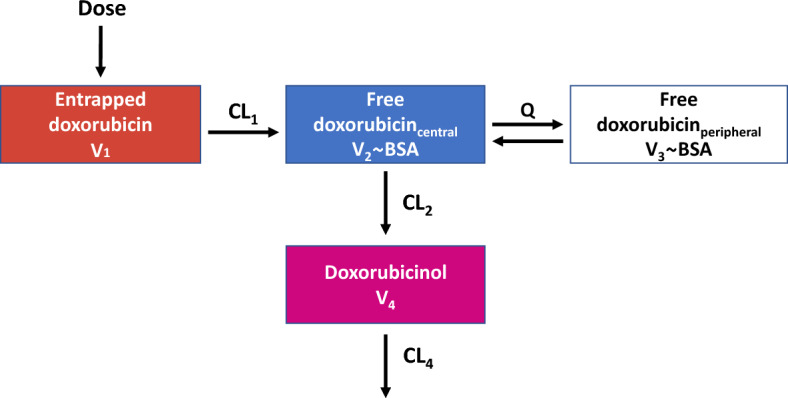
Schematic structure of the joint parent-metabolite PK model of entrapped doxorubicin, free doxorubicin and doxorubicinol. Abbreviations: V1: volume of distribution of entrapped doxorubicin; V2: central volume of distribution of free doxorubicin; V3: peripheral volume of distribution of free doxorubicin; V4: volume of distribution of doxorubicinol; CL1: release clearance of the entrapped doxorubicin; CL2: clearance of free doxorubicin for the metabolism to doxorubicinol; CL4: clearance of doxorubicinol; QDoxo,f: intercompartmental clearance between the central and peripheral compartment of free doxorubicin; BSA: body surface area

In the joint population PK model, the PK of doxorubicin_entrapped_ was best characterised by a one-compartment structural model with central volume of distribution V_1_ (3.39 L, RSE: 7%) and linear clearance CL_1_ (0.0271 L/h, RSE: 10%) to the doxorubicin_free_ compartment. As the clearance parameter for the elimination pathway aiming to capture the removal of liposomes by the RES shrank to zero, it was not retained in the model. For un-encapsulated doxorubicin (doxorubicin_free_), a two-compartment model with central volume of distribution V_2_ (0.531 L at a BSA of 1.75 m^2^, RSE: 16%), peripheral volume of distribution V_3_ (61.3 L at a BSA of 1.75 m^2^, RSE: 19%), intercompartmental clearance Q (0.136 L/h, RSE: 18%), and linear clearance for the metabolism to doxorubicinol CL_2_ (0.450 L/h, RSE: 11%) best characterised the concentration-time profile. For doxorubicinol, a one-compartmental model with central volume of distribution V_4_ (8152 L, RSE: 12%) and linear clearance (CL_4_: 74.6 L/h, RSE: 7%) was sufficient. The final structural model is shown in Fig. 2.

#### Stochastic model

IIV parameters for the structural parameters V_1_, CL_1,_ CL_2_, V_2_, and CL_4_ fulfilled the requirements for model inclusion and were thus retained in the model. As the correlation between the IIV parameter estimates for V_1_ and CL_1_ was high (r = 0.92), a shared-η approach was implemented. IOV was modelled for parameters CL_1_ (14.4% CV, RSE: 12%), V_1_ (8.85% CV, RSE: 10%), CL_2_ (22.4% CV, RSE: 12%) and V_2_ (126% CV, RSE: 10%) and significantly improved model fit. After implementation of IOV on V_2_, the IIV parameter estimate for V_2_ became imprecise and was thus removed from the model without worsening model fit. Implementation of covariances between the other IIV parameters did not improve model fit and was thus not included in the model. The final IIV parameter estimates for CL_1_, V_1,_ CL_2_, and CL_4_ were 45.1% CV (RSE: 11%), 28.2% CV (RSE: 11%), 34.2% CV (RSE 12%), and 15.1% CV (RSE: 10%), respectively. A log-transformed both sides approach with an additive component in the log-domain with separate and uncorrelated parameters for each model species (parameter estimates for doxorubicin_entrapped_, doxorubicin_free_, and doxorubicinol: 19.6% CV (RSE: 5%), 64.2% CV (RSE: 5%), and 65.0% CV (RSE: 7%), respectively) best characterised the residual unexplained variability.

#### Covariate model

BSA was identified as covariate on the central and peripheral volumes of distribution of doxorubicin_free_ V_2_ and V_3_, respectively (V_2__BSA: 4.47, RSE: 19% and V_3__BSA: 11.5, RSE: 18%, Supplementary Fig. 3), significantly improving model fit. A trend of increasing volume of distribution of doxorubicin_entrapped_ with increasing BSA was observed as well, however, implementation of this covariate did not significantly improve model fit. Replacing BSA with other body size descriptors, including body weight or lean body weight, did not improve model fit. No other covariates were identified.

### Model evaluation

**Fig. 3:**
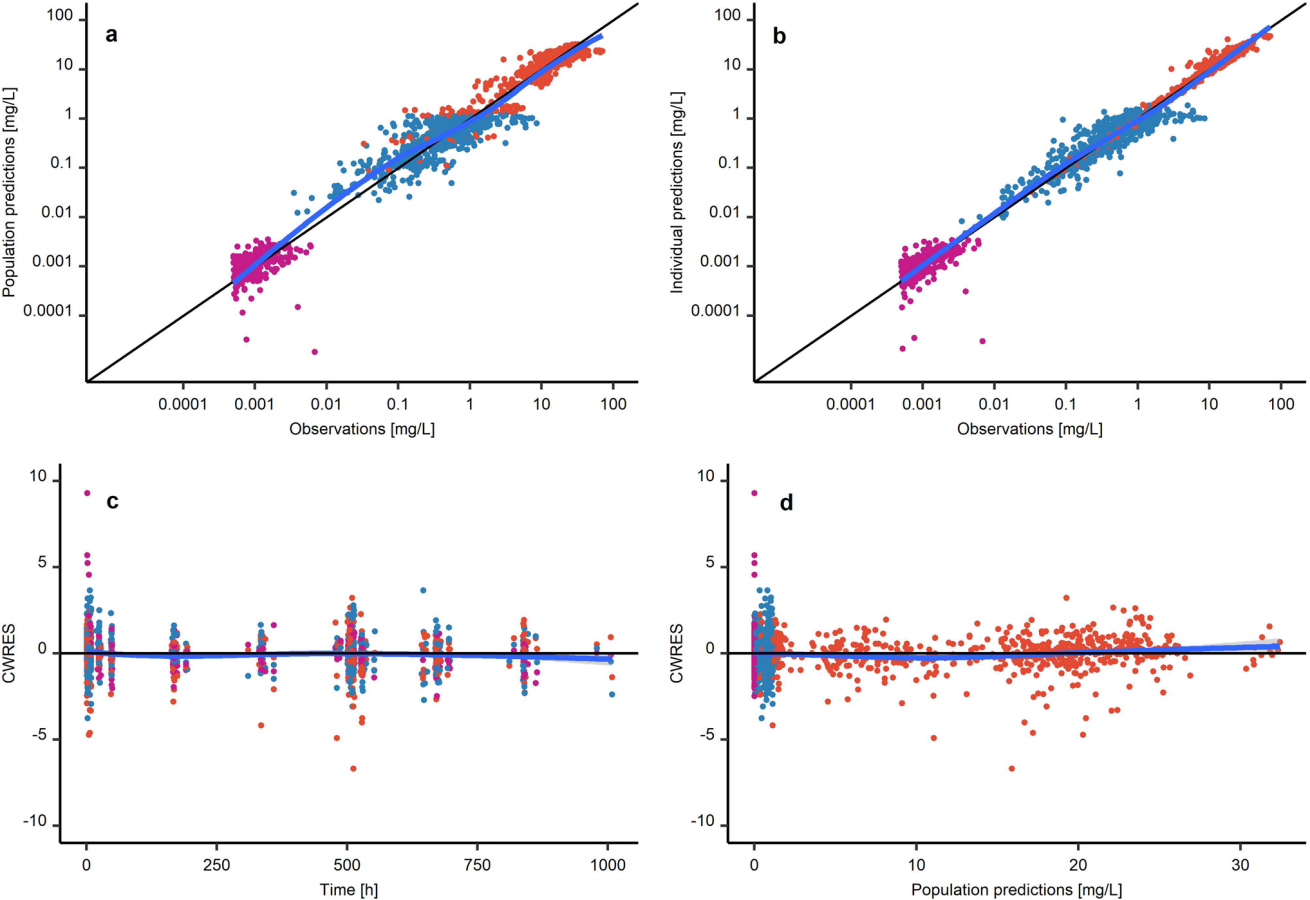
Goodness-of-fit plots of (panel a, upper left) population predictions vs. measured concentrations (“observations”) and (panel b, upper right) individual predictions vs. measured concentrations as well as (panel c, lower left) CWRES vs. Time and (panel d, lower right) CWRES vs. population predictions. Blue lines: trend lines. Red points: entrapped doxorubicin. Blue points: free doxorubicin. Purple points: metabolite doxorubicinol. Abbreviations: CWRES: Conditional weighted residuals

**Fig. 4:**
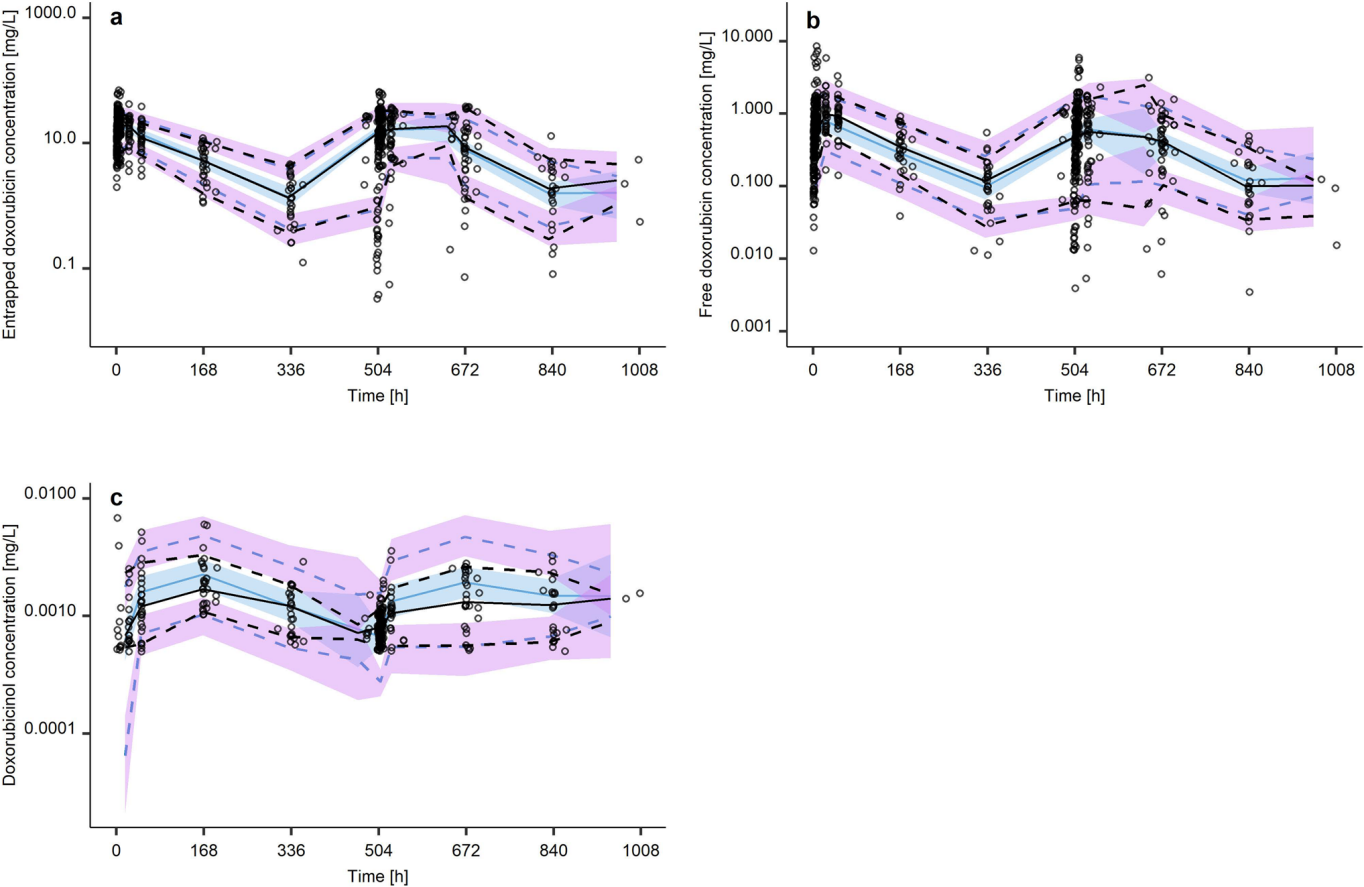
Prediction-corrected visual predictive check for the joint parent-metabolite TLD-1 model characterising entrapped doxorubicin (panel a, upper left), free doxorubicin (panel b, upper right), and doxorubicinol (panel c, lower left). Black solid lines: observed median concentrations; black dashed lines: 10th and 90th percentiles of the observed concentrations; blue solid lines: simulated median concentrations; blue dashed lines: 10th and 90th percentiles of the simulated concentrations; blue shaded areas: 95% confidence intervals around the predicted median concentrations, purple shaded areas: 95% confidence intervals around the 10th and the 90th predicted percentiles, respectively. Black open circles: observed concentrations

The model predictions captured the observed concentrations well (Fig. 3a, b). Furthermore, no systematic bias was identified as indicated by random distributions of conditional weighted residuals vs. time and conditional weighted residuals vs. typical predictions around zero (Fig. 3c, d). Plots overlaying measured concentrations with typical and individual predictions showed a good concordance of predictions and measured concentrations across dose levels and individuals (Supplementary Fig. 4–6). For some measured concentrations, doxorubicin_free_ C_max_ was underpredicted (Supplementary Fig. 5). The final parameter relative standard errors acquired by SIR were low (≤ 19%, Table 2) and the pcVPC showed good predictive model performance (Fig. 4).

**Table 2:** Parameter estimates for the final joint parent-metabolite population pharmacokinetic model of liposomal doxorubicin, free doxorubicin and doxorubicinol using the clinical dataset (n = 30 patients)

Parameter [unit]	Parameter description	Estimate	RSE, %
V_1_ [L]	Volume of distribution of entrapped doxorubicin	3.39	7
CL_1_ [L/h]	Release clearance of entrapped doxorubicin	0.0271	11
V_2_ [L]	Baseline volume of distribution of free doxorubicin	0.531	16
V_2__BSA [-]^b^	Exponent of the power covariate model of BSA on V_2_	4.47	19
Q [L/h]	Intercompartmental clearance of free doxorubicin	0.136	18
V_3_ [L]	Peripheral volume of distribution of free doxorubicin	61.3	19
V_3__BSA [-]^b^	Exponent of the power covariate model of BSA on V_3_	11.5	18
CL_2_ [L/h]	Clearance of free doxorubicin to doxorubicinol	0.450	11
V_4_ [L]	Volume of distribution of doxorubicinol	8152	12
CL_4_ [L/h]	Clearance of doxorubicinol	74.6	7
θ_shared_	Shared η scale factor for V_1_	0.643	10
IIV CL_1_	Interindividual variability in the release of entrapped doxorubicin	45.1% CV	11
IIV V_1_^a^	Interindividual variability in the volume of distribution of entrapped doxorubicin	28.2% CV	11
IIV CL_2_	Interindividual variability in the clearance of free doxorubicin for metabolism to doxorubinol	34.2% CV	11
IIV CL_4_	Interindividual variability in the clearance of doxorubinol	15.1% CV	10
IOV CL_1_	Interoccasion variability for CL_1_	14.4% CV	12
IOV CL_2_	Interoccasion variability for CL_2_	22.4% CV	12
IOV V_1_	Interoccasion variability for V_1_	8.85% CV	10
IOV V_2_	Interoccasion variability for V_2_	126% CV	10
RUV Entrapped doxorubicin	Residual unexplained variability in the observed concentrations of entrapped doxorubicin	19.6% CV	5
RUV Free doxorubicin	Residual unexplained variability in the observed concentrations of free doxorubicin	64.2% CV	5
RUV Doxorubicinol	Residual unexplained variability in the observed concentrations of doxorubicinol	65.0% CV	7

^a^Calculated using a shared-η approach [27] with ω_CL1_·θ_shared_^2^; ^b^implemented as power covariate model, normalised to the median BSA of 1.75 m^2^ using the equation ${V}_{2}\bullet {\frac{BSA}{1.75}}^{V2\_BSA}$ and ${V}_{3}\bullet {\frac{BSA}{1.75}}^{V3\_BSA}$, respectively; *CV*: coefficient of variation; *IIV*: interindividual variability; *IOV*: interoccasion variability; *RUV*: residual unexplained variability; *RSE*: relative standard error = (standard error/estimate) 100

## Discussion

TLD-1 is a novel pegylated liposomal doxorubicin currently in clinical development. Doxorubicin is a widely used antineoplastic drug for the treatment of solid and haematological malignancies, however, its cardiotoxicity is limiting its long-time use [2, 5]. Entrapping it into PEGylated liposomes, such as in Caelyx^®^, virtually eliminates most of its irreversible cardiotoxicity and favourably modifies its PK characteristics. However, the widespread occurrence of PPE during treatment with Caelyx^®^ warrants further efforts to improve the safety of PEGylated liposomal doxorubicin formulations. Additionally, the PK of pegylated liposomal formulations is poorly understood in detail, since it has never been thoroughly studied in dose-escalation studies spanning a large dose range [10, 20, 42].

In this work, we comprehensively characterised the population PK of TLD-1 by non-compartmental analysis and by developing a novel joint parent-metabolite NLME model comprising the three relevant species doxorubicin_entrapped_, doxorubicin_free_, and metabolite doxorubicinol, based on densely sampled patients over a 4.5-fold dose range. Our model predictions captured the measured concentrations well, however, for 10 patients, the initial peak doxorubicin_free_ concentrations were underestimated. The reason for this is yet to be elucidated – it could be hypothesised that for these patients, a fraction of doxorubicin had already been released from the liposomes at the time of infusion. This would lead to an unexpectedly high peak in doxorubicin_free_ under the assumption that all doxorubicin was entrapped at the time of infusion. However, this hypothesis is not supported by in-house manufacturer data showing that > 99% of doxorubicin is entrapped in the liposomes in the drug product. Furthermore, during model development, we attempted to extend our model based on this hypothesis by estimating the fractions of TLD-1 dose being entrapped and free at time of infusion. However, we estimated a fraction of 99% to be entrapped at the time of infusion, supporting independent stability data from the manufacturer. Furthermore, the described model extension did not improve model predictions. As it additionally inflated the model run time and parameter estimate imprecision, we did not include it in the final model by assuming all drug being encapsulated at the time of infusion. Additional exploratory analysis did not reveal any correlations between any of the patient characteristics available in our dataset and the doxorubicin_free_ concentrations. Further research should thus focus on the occurrence of unexpectedly high initial concentrations of doxorubicin_free_. Our model parameter estimates, e.g., the estimate for the volume of distribution of doxorubicin_entrapped_ V_1_: 3.39 L, were plausible and in line with previously reported volumes of distribution of pegylated liposomal doxorubicin (median: 3.90 L, range: 2.10–10.0 L) [10].

Furthermore, we identified and quantified different levels of variability such as IIV, IOV and RUV. Both IIV and IOV were estimated to be moderate for all parameters (≤42.6% CV) except for the IOV on the volume of distribution for doxorubicin_free_ V_2_ (IOV V_2_: 125% CV). A possible explanation for this high variability between cycles could be the fast elimination of doxorubicin compared to the relatively slow release of doxorubicin from the liposomes (0.450 L/h vs. 0.0271 L/h). Thus, the distribution time of doxorubicin_free_ between release and elimination is small and a robust estimation of its volume of distribution challenging. The estimated release rate of doxorubicin from the liposomes (TLD-1: CL_1_: 0.0271 L/h, corresponding to a leakage half-life of 86.7 h at the estimated liposomal volume of distribution of 3.39 L) was lower than the previously published leakage half-life of doxorubicin from Caelyx^®^ liposomes (118.4 h) [17]. In general, a longer leakage half-life has been associated with better efficacy [17]. Interestingly, the median half-life of total doxorubicin was longer in TLD-1 compared to Caelyx^®^ (95 h vs. 71.5 h [8]). The increased half-life could be due to the novel liposome manufacturing process used for TLD-1, ensuring the localisation of PEG only on the outer layer of the liposomes and leading to more effective protection of the liposomes from the RES. Significant clinical correlations between longer half-lives, smaller clearance, and higher dose-normalised AUC with longer survival have been observed in a clinical study on the PK of mitomycin-entrapped liposomes [43]. Thus, a correlation of half-life with efficacy could also be investigated for TLD-1 in the future. Moreover, the available half-life data could aid in the continued development of the compound by optimising the dosing interval(s).

Our thorough PK characterisation of a seven dose level, 4.5-fold range dataset, including the free drug and the main metabolite at the population (typical parameter values) as well as on the individual level (considering the different variability components) allows to explore potential links of plasma concentrations with clinical outcome and toxicity data of the phase I trial [44] next. These future investigations shall focus on exploratory exposure-response and exposure-toxicity relationships and, most importantly, clinical correlations between the estimated PK parameters and outcome. As the IIV for the doxorubicin release efficiency CL_1_ was moderate (45.1% CV) and the IOV lower (14.4% CV), predicting the probability for treatment success by calculating individual CL_1_ based on individual PK samples should be investigated in the future. If successful, this could be then applied in individualised dosing [45]. Our model can additionally be used in various model-informed drug development applications, such as in clinical trial simulations or in optimal design analysis, informing future clinical study designs.

In summary, TLD-1 is a new compound aiming to further improve efficacy and reduce toxicity of pegylated liposomal doxorubicin. In this work, we presented the thorough PK data analysis of TLD-1, which is currently in continuing clinical development. The developed joint parent-metabolite NLME model can now be integrated with recently published efficacy and toxicity data [44] to explore potential exposure-response relationships.

## Supplementary Information

Below is the link to the electronic supplementary material.

Supplementary file1 (DOCX 2202 KB)

## Data Availability

The datasets generated during and/or analysed for the presented study are available from the corresponding authors on reasonable request.
