# Supplementary material for: Population pharmacokinetics of TLD-1, a novel liposomal doxorubicin, in a phase I trial
Source: Cancer Chemother Pharmacol. 2024 Jun 15;94(3):349–60. doi: 10.1007/s00280-024-04679-z (PMC11420315; doi:10.1007/s00280-024-04679-z)
Supplement: Supplementary file 1 — Supplementary file1 (DOCX 2202 KB) [file 280_2024_4679_MOESM1_ESM.docx]

# Supplementary Information

**Journal name:** Cancer Chemotherapy and Pharmacology

# Population pharmacokinetics of TLD-1, a novel liposomal doxorubicin, in a phase I trial

Anna M. Mc Laughlin^a,b#^, Dagmar Hess^c#^, Robin Michelet^a^, Ilaria Colombo^d^, Simon Haefliger^e^, Sara Bastian^f^, Manuela Rabaglio^e^, Michael Schwitter^g^, Stefanie Fischer^c^, Katrin Eckhardt^h^, Stefanie Hayoz^h^, Christoph Kopp^h^, Marian Klose^a,b^, Cristiana Sessa^d^, Anastasios Stathis^i,j^, Stefan Halbherr^k^, Wilhelm Huisinga^l^, Markus Joerger^c*†^, Charlotte Kloft^a*†^

^a^Dept. of Clinical Pharmacy and Biochemistry, Institute of Pharmacy, Freie Universitaet Berlin, Kelchstr. 31, 12169 Berlin, Germany

^b^Graduate Research Training Program PharMetrX, Freie Universitaet Berlin/University of Potsdam, Berlin/Potsdam, Germany

^c^Department. of Medical Oncology and Haematology, Cantonal Hospital St. Gallen, St. Gallen, Switzerland

^d^Department of Medical Oncology, Oncology Institute of Southern Switzerland, EOC, Bellinzona, Switzerland

^e^Department of Medical Oncology, Inselspital, Bern University Hospital, University of Bern, Bern, Switzerland

^f^Department of Medical Oncology, Kantonsspital Graubünden, Chur, Switzerland

^g^Oncology/Hematology, Kantonsspital Graubünden, Chur, Switzerland

^h^Coordinating Center, Swiss Group for Clinical Cancer Research, Bern, Switzerland

^i^Oncology Institute of Southern Switzerland, EOC, Bellinzona, Switzerland

^j^Faculty of Biomedical Sciences, Universita della Svizzera Italiana, Lugano, Switzerland

^k^Innomedica Switzerland AG, Switzerland

^l^Institute of Mathematics, University of Potsdam, Potsdam, Germany

^#^Shared first authorship ^†^Shared senior authorship

*Corresponding authors:

Prof. Dr. Charlotte Kloft
Email: [charlotte.kloft@fu-berlin.de](mailto:charlotte.kloft@fu-berlin.de)
Freie Universität Berlin
Institut für Pharmazie
Abteilung Klinische Pharmazie & Biochemie
Kelchstr. 31
12169 Berlin
Germany

Prof. Dr. Dr. Markus Joerger
Email: [markus.joerger@kssg.ch](mailto:markus.joerger@kssg.ch)
Kantonsspital St. Gallen
Klinik für Medizinische Onkologie und Hämatologie
Rorschacher Strasse 95
9007 St. Gallen
Switzerland


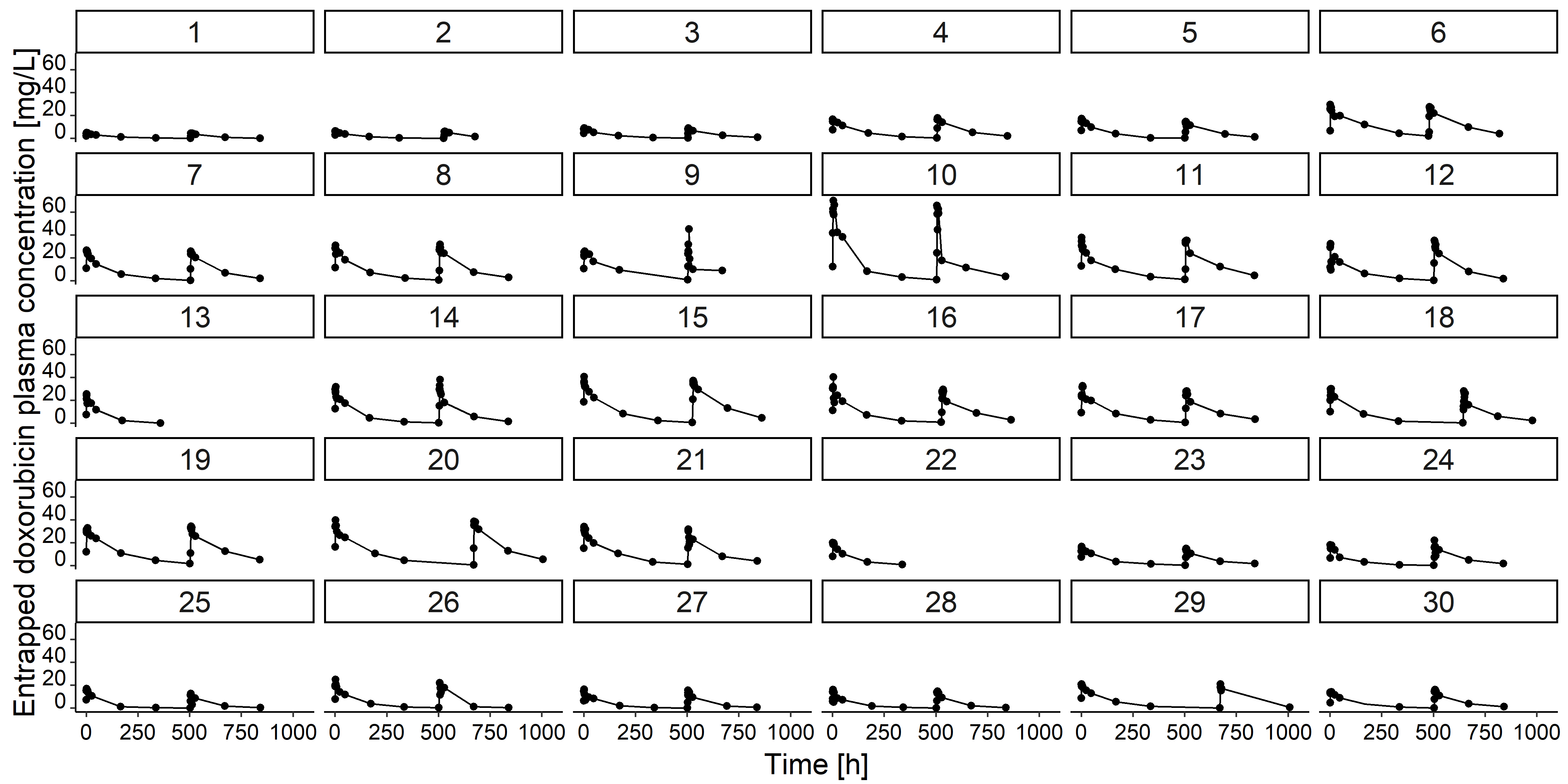


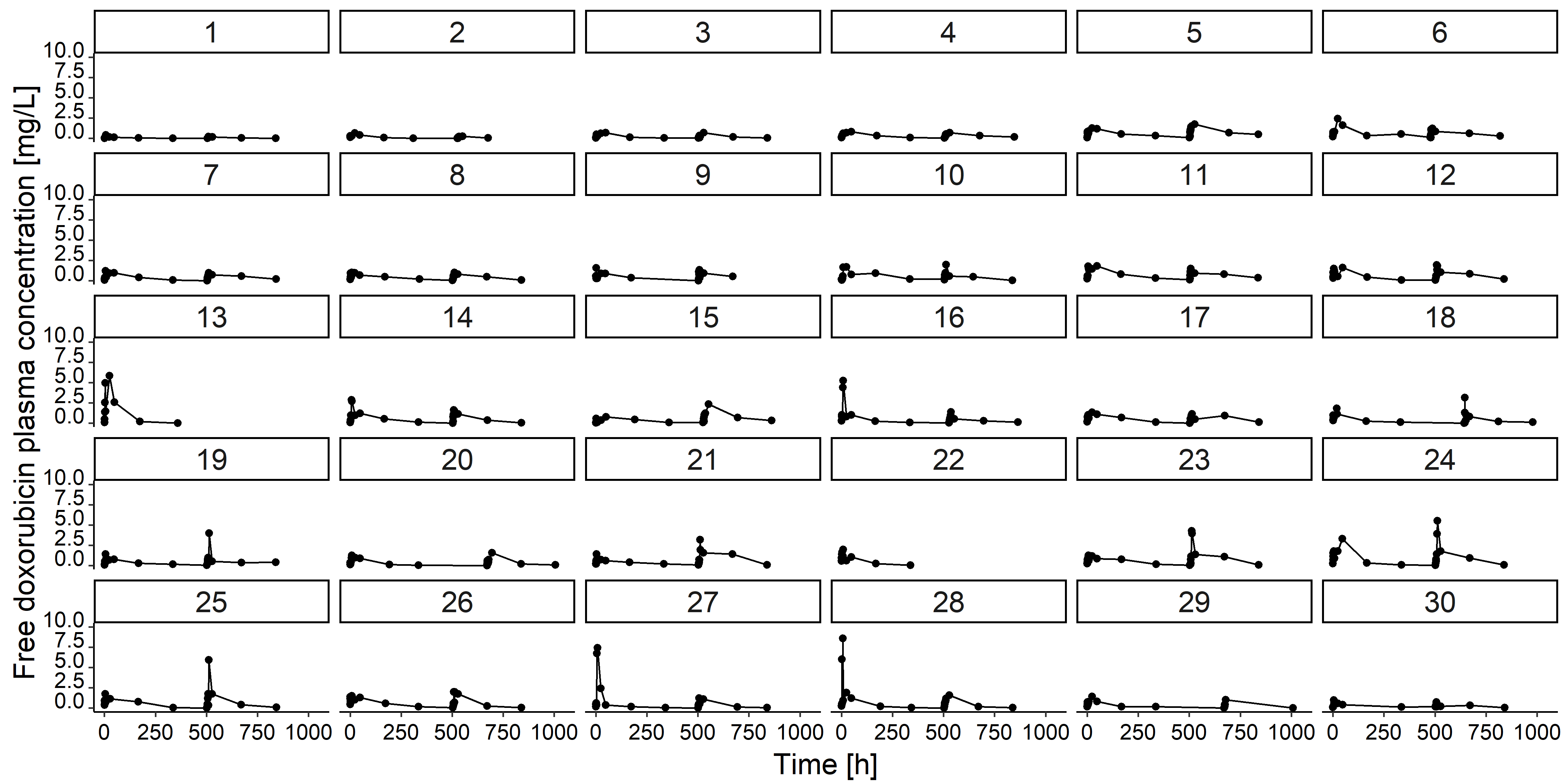


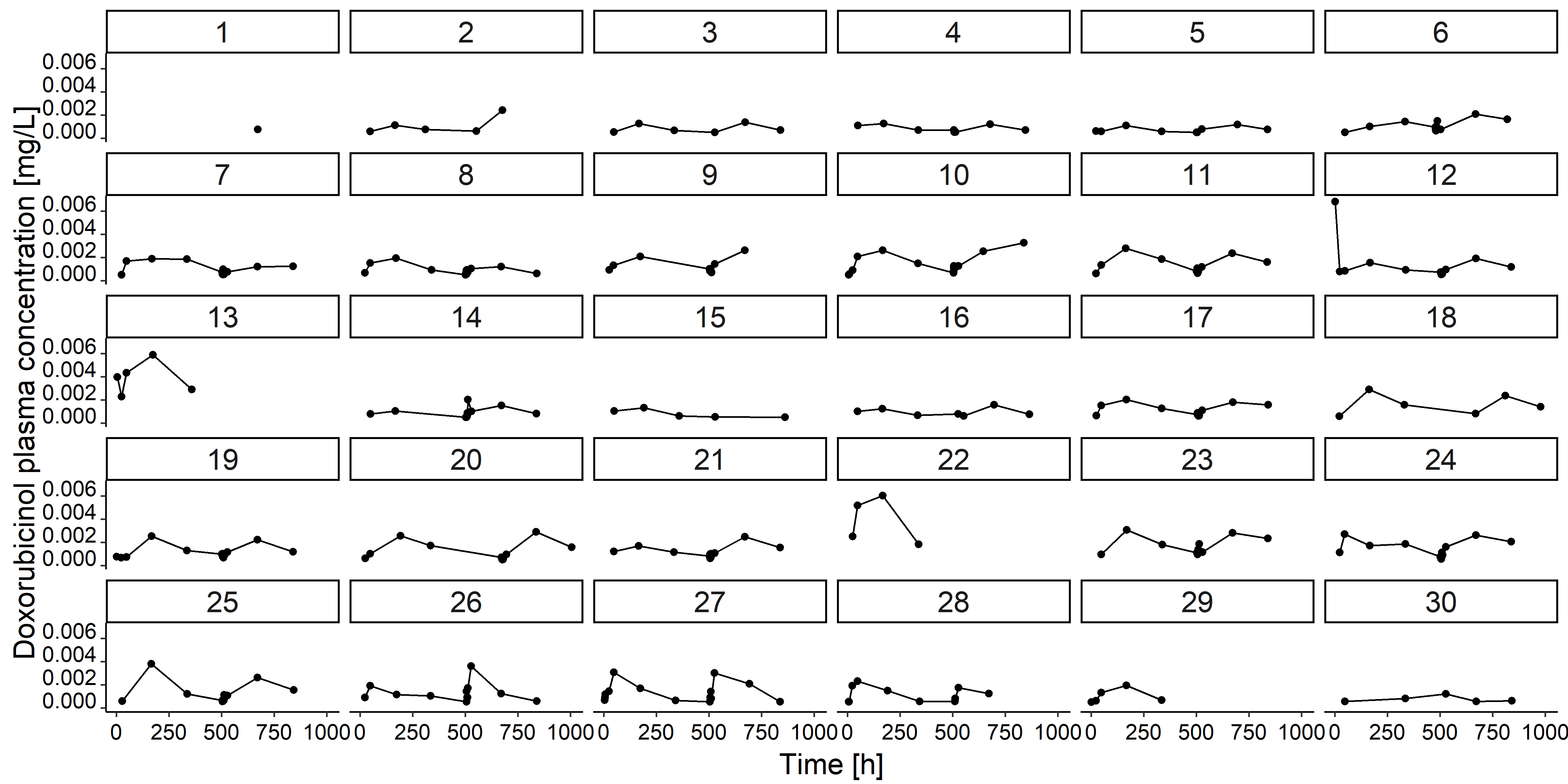


**Supplementary Fig. 1** Concentration-time profiles of the observed plasma concentrations of entrapped doxorubicin (upper plots), free doxorubicin (middle plots), and doxorubicinol (lower plots) in the clinical TLD-1 dataset. Each panel represents an individual patient.


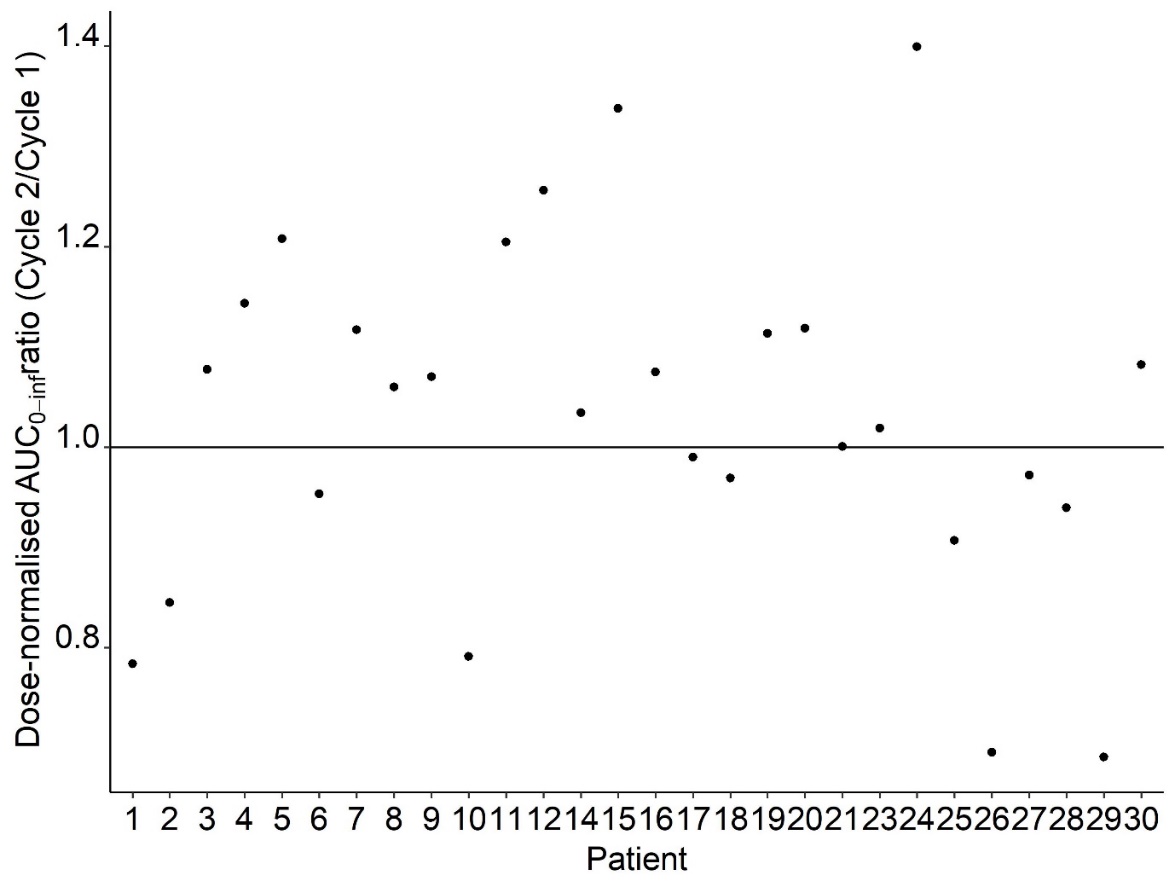


**Supplementary Fig. 2** Ratios of individual dose-normalised AUC_0-inf_ of total doxorubicin in cycle 1 vs 2 in 28 patients treated with TLD-1. Two of the 30 patients were not included in this plot because for these patients only data from one cycle was available. *Abbreviations:* AUC_0-inf_: Area under the concentration-time curve from t=0 to infinity.


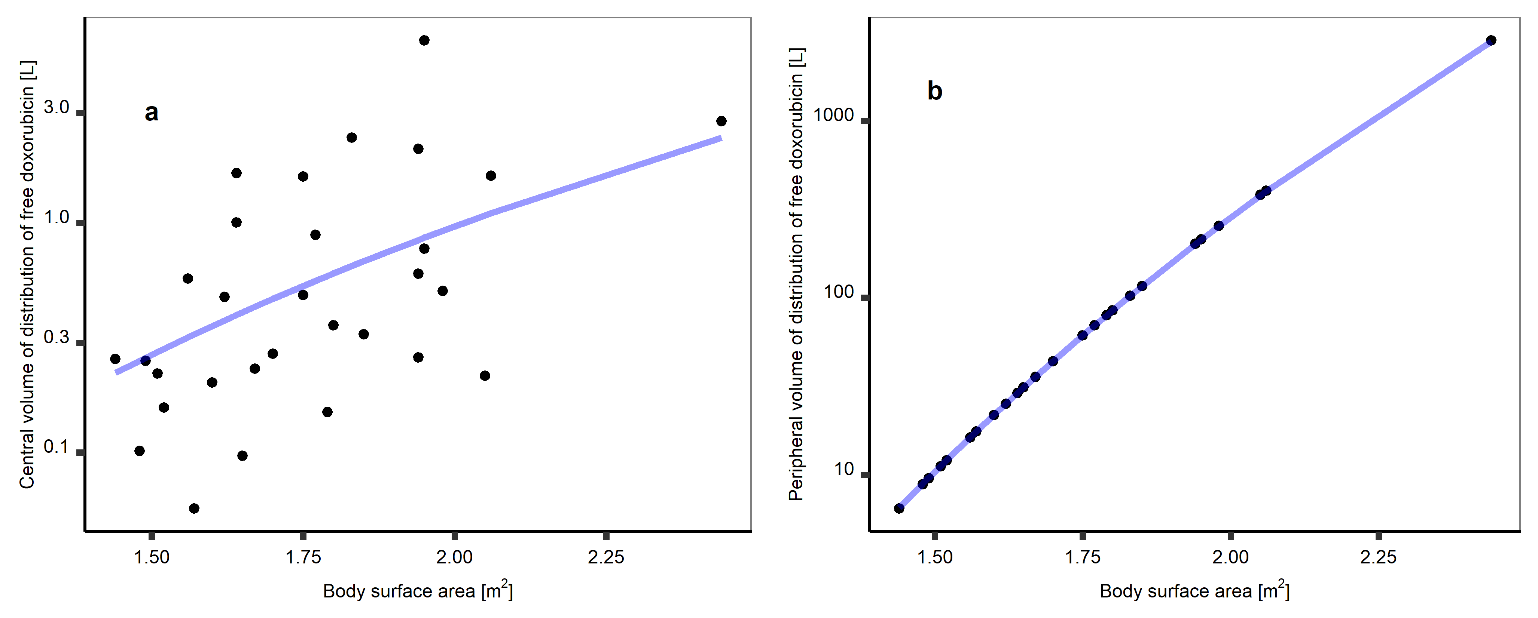


**Supplementary Fig. 3** Individual model-predicted central (panel a, left) and peripheral (panel b, right) volumes of distribution of doxorubicin_free_ vs. body surface area. Blue lines: typical model predictions for the central and peripheral volumes of distribution of doxorubicin_free_ vs. body surface area.


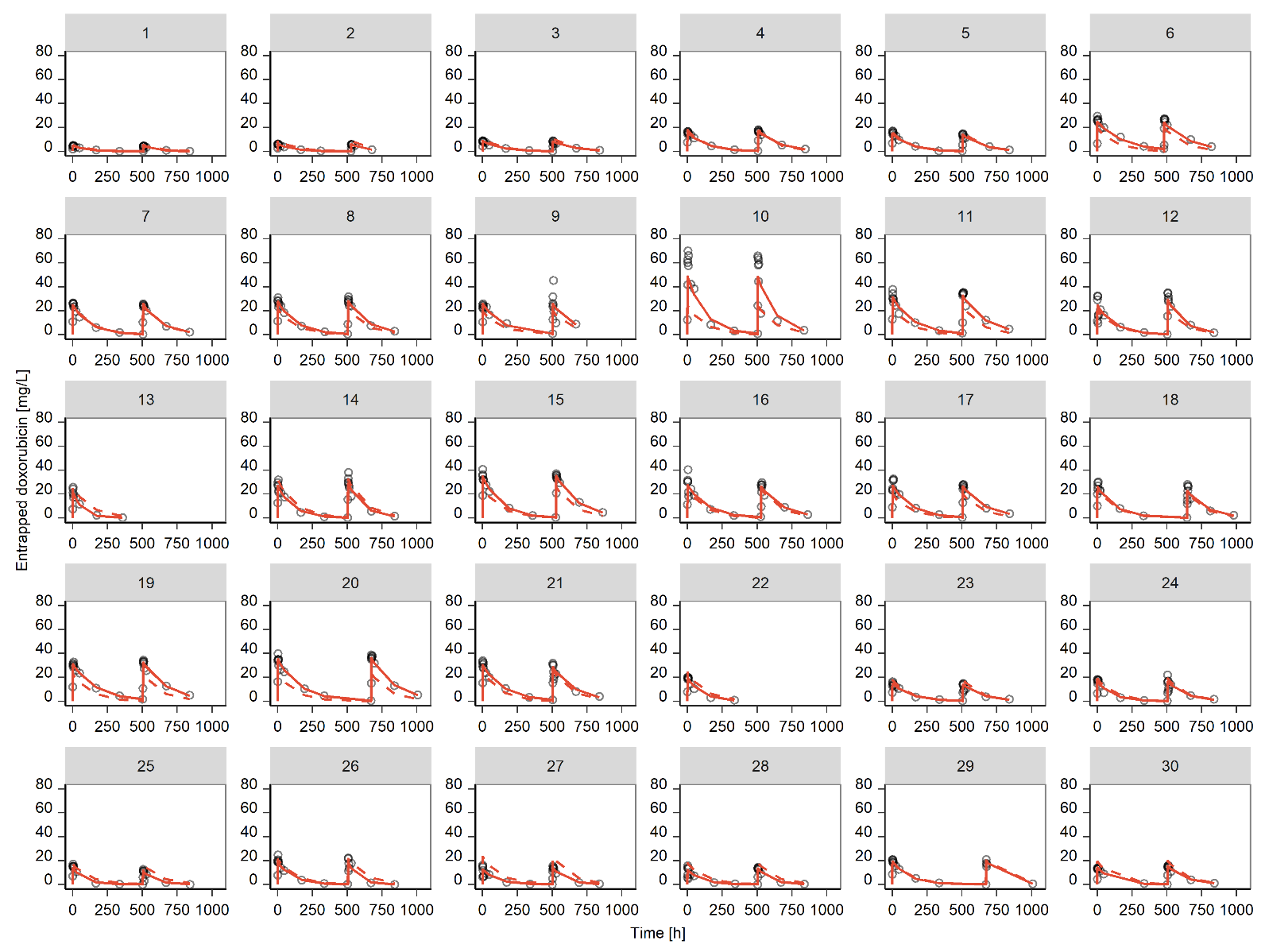


**Supplementary Fig. 4** Entrapped doxorubicin: Measured concentrations (*data points*), typical predictions (*dashed line*s) and individual predictions (*solid lines*) in the clinical TLD-1 dataset.


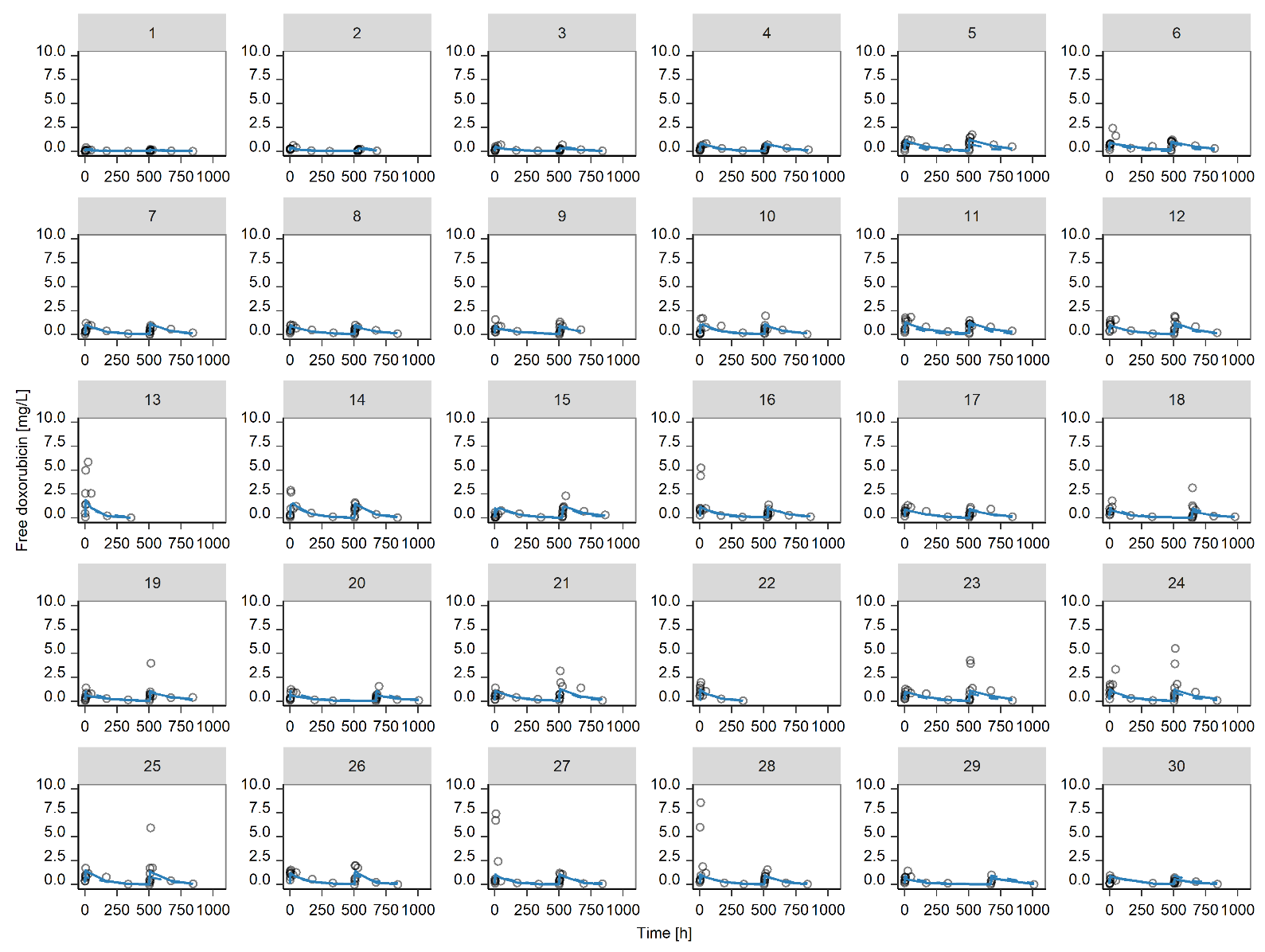


**Supplementary Fig. 5** Free doxorubicin: Measured concentrations (*data points*), typical predictions (*dashed lines*) and individual predictions (*solid lines*) in the clinical TLD-1 dataset.


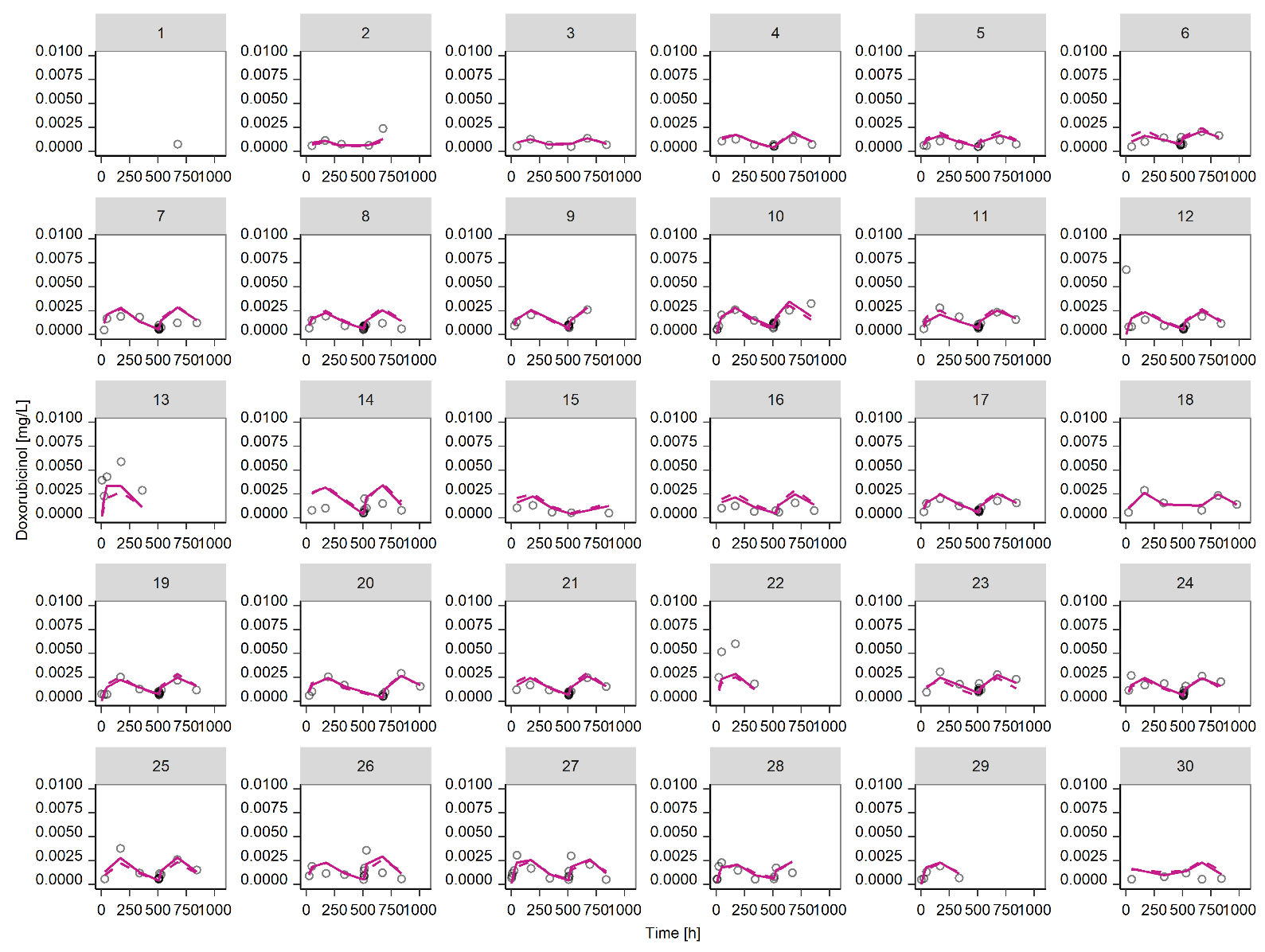


**Supplementary Fig. 6** Doxorubicinol: Measured concentrations (*data points*), typical predictions (*dashed lines*) and individual predictions (*solid lines*) in the clinical TLD-1 dataset.
